# Early detection of structural abnormalities and cytoplasmic accumulation of TDP-43 in tissue-engineered skins derived from ALS patients

**DOI:** 10.1186/s40478-014-0181-z

**Published:** 2015-01-31

**Authors:** Bastien Paré, Lydia Touzel-Deschênes, Rémy Lamontagne, Marie-Soleil Lamarre, François-Dominique Scott, Hélène T Khuong, Patrick A Dion, Jean-Pierre Bouchard, Peter Gould, Guy A Rouleau, Nicolas Dupré, François Berthod, François Gros-Louis

**Affiliations:** Department of Surgery, Faculty of Medicine, Laval University, Québec, Canada; CHU de Québec Research Center, LOEX-Hôpital de l’Enfant-Jésus, 1401, 18e rue, Quebec, G1J 1Z4 Canada; ALS Clinic, Department of Neurological Sciences, CHU de Québec and the Faculty of Medicine, Laval University, Québec, Canada; Montreal Neurological Institute and Hospital, Department of Neurology and Neurosurgery, McGill University, Montréal, Canada; Department of Medical Biology, Division of Anatomic Pathology and Neuropathology, CHU de Québec, Hôpital de l’Enfant-Jésus, Québec, Canada

**Keywords:** Amyotrophic Lateral Sclerosis (ALS), TDP-43, Biomarker, Tissue engineering, Skin, Neuropathology, Extracellular matrix

## Abstract

Amyotrophic lateral sclerosis (ALS) is an adult-onset disease characterized by the selective degeneration of motor neurons in the brain and spinal cord progressively leading to paralysis and death. Current diagnosis of ALS is based on clinical assessment of related symptoms. The clinical manifestations observed in ALS appear relatively late in the disease course after degeneration of a significant number of motor neurons. As a result, the identification and development of disease-modifying therapies is difficult. Therefore, novel strategies for early diagnosis of neurodegeneration, to monitor disease progression and to assess response to existing and future treatments are urgently needed. Factually, many neurological disorders, including ALS, are accompanied by skin changes that often precede the onset of neurological symptoms. Aiming to generate an innovative human-based model to facilitate the identification of predictive biomarkers associated with the disease, we developed a unique ALS tissue-engineered skin model (ALS-TES) derived from patient’s own cells. The ALS-TES presents a number of striking features including altered epidermal differentiation, abnormal dermo-epidermal junction, delamination, keratinocyte infiltration, collagen disorganization and cytoplasmic TDP-43 inclusions. Remarkably, these abnormal skin defects, uniquely seen in the ALS-derived skins, were detected in pre-symtomatic *C9orf72*-linked ALS patients carrying the GGGGCC DNA repeat expansion. Consequently, our ALS skin model could represent a renewable source of human tissue, quickly and easily accessible to better understand the physiopathological mechanisms underlying this disease, to facilitate the identification of disease-specific biomarkers, and to develop innovative tools for early diagnosis and disease monitoring.

**Electronic supplementary material:**

The online version of this article (doi:10.1186/s40478-014-0181-z) contains supplementary material, which is available to authorized users.

## Introduction

Among neurological disorders, neurodegenerative diseases are becoming more and more prominent because of their severity and increasing frequency in aging populations. At present, the causes and pathogenic mechanisms of these diseases remain largely unknown. In addition, these diseases are currently incurable and difficult to diagnose before a late stage due to lack of efficient biomarkers or good human-based models to study them. Amyotrophic lateral sclerosis (ALS) is an adult-onset neurodegenerative disease characterized by the selective degeneration of motor neurons in the brain and spinal cord resulting in progressive paralysis and death [1]. The lifetime risk to develop the disease is approximately 1 in 1000 [1]. The median survival is approximately 2 years from diagnosis and 3 years from symptom onset. To date, there is no treatment that will meaningfully alter the course of this disease.

Current diagnosis of ALS is based on clinical assessment of related symptoms (Additional file 1: Table S1) [2]. The clinical manifestations observed in ALS appear only after degeneration of a significant number of motor neurons. As a result, the identification and development of disease-modifying therapies is difficult, making ALS an incurable disease and a serious challenge for neurologists. The latency between the first symptoms and a formal diagnosis of ALS has remained unchanged for more than a decade and ranges from 8.0 to 15.6 months [2]. At such a late stage, a large proportion of motor neurons have already been lost. Therefore, novel discoveries of sensitive and specific biomarkers for ALS are needed to facilitate diagnosis at early stages, monitor disease progression, and assess response to existing and future treatments.

In the initial stages, ALS is characterized predominantly by a heterogeneous presentation of symptoms. Most patients display cramps, weakness and muscle atrophy of the hands and feet progressing to the forearms, shoulder and legs eventually leading to complete paralysis. Some patients have their bulbar muscles primarily affected, influencing speech and swallowing. In the majority of cases, cognitive functions are relatively intact with subtle cognitive changes being observed in 30-50% of cases; albeit comorbidity between frontotemporal dementia (FTD) and ALS is observed in 25% of cases [3]. While motor neuron degeneration remains the central component, there is considerable phenotypic variability including site of onset, survivorship and the presence or absence of cognitive impairment. ALS can thus be justifiably considered a heterogeneous disorder sharing in common the degeneration of motor neurons. This syndrome is currently unified by the post mortem finding of ubiquitinated TDP-43 cytoplasmic inclusions in motor neurons of the central nervous system (CNS) in post-mortem human pathologic tissues, a pathological hallmark now commonly found in the majority of familial ALS (FALS) cases with or without *TARDBP* mutations, and in sporadic ALS (SALS) cases [4-14]. In healthy motor neurons, TDP-43 is typically localized to the nucleus. TDP-43 thus becomes a potential marker associated with the vast majority of ALS cases. At the moment, since cytoplasmic TDP-43 inclusions are only found in post-mortem CNS tissues, there are two major limitations of using TDP-43 as a potential biomarker of the disease. First, brain and spinal cord biopsies are too invasive and as a consequence longitudinal studies become impossible. Secondly, the different sites of onset, as well as the heterogenous spreading, make it difficult to standardize the choice of the site of biopsy.

The non-cell autonomous toxicity paradigm in ALS has been well establish as they are increasing evidences that it is the convergence of damage developed within multiple cell types, including within neighboring non-neuronal supporting cells, which is crucial to neuronal dysfunction in ALS [15-21]. The involvement of other cell types reveals certainly new perspectives to better understand this disease. Due to the common embryonic origin of both skin and neural tissue from the ectodermal germ layer, many neurological disorders, including ALS, are accompanied by skin changes that often precede the apparition of neurological symptoms [22]. Aiming to generate an innovative human-based model and to identify predictive biomarkers associated with the disease, we developed a unique ALS tissue-engineered skin model (ALS-TES), derived from patient’s own cells. Our results show that our ALS-TES present a number of striking features uniquely seen in ALS-derived skins.

The identification of biomarkers in ALS has been a very active area of investigation, employing transcriptional studies, protein profiling in blood and CSF, imaging, and electrophysiological techniques [23]. While these techniques have identified some potential ALS biomarkers, so far none have proven to be clinically useful. Our results show that it is possible to detect a number of abnormal features associated with ALS using our tissue-engineered skin model. Consequently, our ALS-TES model could represent a renewable source of human tissue, quickly and easily accessible to better understand the physiophatological mechanisms underlying these diseases, to identify predictive disease biomarker and hopefully to develop innovative tools for disease monitoring and drug screening.

## Materials and methods

### Patients

Cases were recruited through the designated ALS clinics in Quebec (Drs Dupré and Rouleau). Every index case met the El Escorial criteria for clinically definite, probable or laboratory supported ALS (Additional file 1: Table S1). All cases signed a consent form approved by our Institutional Ethics Committees (Comité d'éthique de la recherche du CHU de Québec) prior to being enrolled in the study and were recruited on a voluntary basis. Skin biopsies were collected from affected and unaffected individuals (Table 1). Besides collecting skin biopsies, blood samples were also collected and used for DNA extraction and patient’s genotyping. In totals 6 SALS (Table 1), 6 FALS-linked *C9orf72* patients (Table 1 and Additional file 2: Figure S1) and 6 control individuals were recruited. Relative controls, matching for both environmental exposures and genetic background as well as with socio-economic status, ethnicity and age, have been also recruited for this study. This type of controls represents the perfect group in terms of matching for age, sex ratio, ethnicity and environmental exposures [24].

**Table 1:** **Data information on ALS patients and controls recruited in the study**

**ID number**	**Biopsy location**	**Sex**	**Age at sampling**	**Current age**	**Age of death**	**Clinical status**	**Genetic status**
SALS 1	Arm	M	48	NA	49	Affected	No known ALS-associated mutations
SALS 2	Arm	M	34	NA	34	Affected	No known ALS-associated mutations
SALS 3	Arm	M	64	NA	64	Affected	No known ALS-associated mutations
SALS 4	Arm	F	54	NA	54	Affected	No known ALS-associated mutations
SALS 5	Arm	M	59	NA	59	Affected	No known ALS-associated mutations
SALS 6	Arm	F	58	NA	58	Affected	No known ALS-associated mutations
C9-S000005	Arm	F	63	65	NA	Clinically unaffected	*C9orf72* expansion
C9-S000008	Arm	F	47	49	NA	Clinically unaffected	*C9orf72* expansion
C9-S000009	Arm	F	59	60	NA	Affected	*C9orf72* expansion
C9-S000012	Arm	M	52	54	NA	Clinically unaffected	*C9orf72* expansion
C9-S000013	Arm	F	49	51	NA	Clinically unaffected	*C9orf72* expansion
C9-S000014	Arm	M	46	47	NA	Clinically unaffected	*C9orf72* expansion
Ctrl 1	Arm	F	55	NA	NA	Control	NA
Ctrl 2	Arm	F	48	NA	NA	Control	NA
Ctrl 3	Arm	M	50	NA	NA	Control	NA
Ctrl 4	Arm	M	62	NA	NA	Control	NA
Mui638x	Arm	F	38	NA	NA	Control	NA

### Skin biopsies and cell extraction/culture

For each participant, two skin biopsies were collected using a 6-mm diameter punch biopsy. All skin biopsies were taken from the same body area for each subject. Skin cells (fibroblasts and keratinocytes) were isolated in order to generate tissue-engineered skin derived from each cases and subjects as previously described [25]. Briefly, the skin biopsies were incubated with 0,05% thermolysine (Sigma, Oakville, QC, Canada), overnight at 4°C, in order to facilitate the mechanical separation of the epidermis from the dermis. Then, fibroblasts and keratinocytes were isolated from the dermis and the epidermis respectively after treatment with 0,2 IU/mL collagenase H (Roche, Missisauga, Ontario, Canada) and with 0,05% trypsin (Intergen lot: xt20012, New-York, USA), 0,01% EDTA (J.T. Baker, Center Valley, PA, USA). Cultured fibroblasts, grown in DMEM (Dulbecco-Vogt modification of Eagle's medium) (Invitrogen, Burlington, ON, Canada) supplemented with 10% Fetal Calf Serum (FCS) (Invitrogen), 100 IU/ml penicillin G (Sigma, Oakville, QC, Canada) and 25 μg/ml gentamicin (Schering, Pointe-Claire, QC, Canada) in 8% CO2 at 37°C, at passage five were seeded at a concentration of 1,5 × 10^5^ cells on tissue culture dishes. Keratinocytes were seeded at 8 × 10^5^ cells on a feeder layer of irradiated 3T3 mouse fibroblasts and were cultured in a combination of Dulbecco-Vogt modification of Eagle's medium with Ham's F12 (3:1) supplemented with 5% Fetal Clone II serum (Hyclone, Scarborough, Ontario, Canada), 5 μg/mL insulin (Sigma Oakville, Canada), 0.4 μg/mL hydrocortisone (Calbiochem, EMD Biosciences, Gibbstown, NJ), 10 − 10 M cholera toxin (MP Biomedicals, Montréal, Québec, Canada), 10 ng/mL human epidermal growth factor (Austral Biological, San Ramon, CA), 100 IU/mL penicillin G (Sigma), and 25 μg/mL gentamicin (Schering).

### Production of tissue-engineered skin equivalents

At confluence, fibroblast medium was supplemented with 50 μg/ml of ascorbic acid (Sigma, Oakville, Qc, Canada) for 28 days in order to induce secretion of extracellular matrix (ECM) proteins and to form a fibroblast sheet. Three fibroblast sheets were superimposed and cultured for 7 additional days to allow sheet adhesion. Isolated keratinocytes at passage three were seeded on top of the mature fibroblast sheets at a concentration of 1 × 10^5^ cells to form a new epidermal layer. The reconstructed skin equivalent was then cultivated for another week prior to cultivate the skin equivalents at the air-liquid interface in order to enhance formation of the *stratum corneum* (outermost layer of the epidermis) for two other weeks. See Additional file 3: Figure S2 for more details.

### Autopsy procedure

The autopsy procedure in brief is that the spinal vertebrae are exposed by a standard dorsal approach and then sectioned through the vertebral foramina. The posterior vertebral arcs are then removed *en bloc* and the dural sac exposed. The spinal cord is sectioned in the mid cervical region to avoid further dissection of the neck. The spinal cord from mid-cervical region to filum terminale is then removed within the dural sac with short segments of peripheral nerve and spinal ganglia. The mid and lower cervical cord is often submitted for research and directly stored at -80°C. Further neuropathologic examination occurs after fixation of the thoracic, lumbar and sacral spine within the dural sac in a dedicated chamber. The dural sac is pinned at either end to prevent retraction during fixation.

### Immunohistochemistry (IHC)

IHC was performed on 5-μm thick sections subsequently fixed in 4% paraformaldehyde dissolved in phosphate buffer for 2 h at room temperature or overnight at 4°C. Sections were first quenched in 0.3% H_2_O_2_ for 30 min, permeabilized in 1% Tween 20 for 10 min, blocked in 5% normal goat serum for 1 h, and then incubated with the primary rabbit anti-TDP43 antibody (cat# 12892-1-AP, 1:1000, Proteintech Group Inc., Chicago, IL, USA) for 16 h at 4°C. Visualization was made by incubating the slides with a biotinyled goat anti-rabbit secondary antibody coupled to horseradish peroxidase (1:500, Jackson Immunoresearch Laboratories, West Grove, PA, USA). Please note that the commercial TDP-43 antibody used in this study recognizes the cleavage product of 20-30 kDa in addition to the native and phosphorylated forms of TDP-43.

### Masson’s trichrome staining

For Masson’s trichrome staining, ALS-TES were fixed in Histochoice (Amresco, Solon, OH) and embedded in paraffin. Microtome sections (5-um thick) were stained with Masson’s trichrome using Weigert’s hematoxylin, fuchsin-ponceau, and aniline blue stains. Deparaffinization of the tissues were done by incubating the slides in toluene (Chaptec, Pointe-Aux-Trembles, Québec, Canada) for 10 minutes (2 times 5 minutes) followed by 20 quick in and out dips in 99% ethanol (Les Alcools Commerciales, Brampton, Ontario, Canada). Slides were then incubated for 10 minutes in Weigert's Hematoxylin (Millipore, Darmstadt, Germany) and washed for 5 minutes under running tap water. Weigert's Hematoxylin solution was prepared as followed: 200 ml of solution 1 (Millipore, Cat. 7341×-71), 200 ml of solution 2 (Millipore, Cat. 7342×-71) and 1 ml of chlorhydric acid 2 N (Fisher Scientific, Ottawa, Ontario). A second incubation in Fuchsin-Ponceau stain solution for 5 minutes followed by 10 quick in and out dips in 0,1% acetic acid (diluted in water) was also performed. Fuchsin-Ponceau solution was prepared as followed: 400 mL ddH2O, 0,13 g fuchsine acid (Sigma, Cat. F-8129), 0,27 g xylidine ponceau (Sigma, Cat. P2395) and 0,8 mL glacial acetic acid (J.T. Baker, Center Valley, PA, USA, Cat. 9508). Slides were then incubated in freshly made 5% phosphomolybdic acid for 6 minutes followed by 10 quick in and out dips in 0,1% acetic acid (diluted in water) prior another series of 10 dips in distilled water. Phosphomolybdic acid solution was prepared as followed: 200 mL ddH2O, 10 g phosphomolybdic acid (Alfa aesar, Ward Hill, MA, USA, Cat. 56166). A final incubation in aniline blue for 5 minutes followed by 10 quick dips 0,1% acetic acid (diluted in water) and 10 other quick in and out dips in ethanol 99% was also carried out. Aniline blue solution was prepared as followed: 400 mL of boiling ddH2O, 4 g aniline blue (Fisher Scientific, Ottawa, Ontario, Cat. A967), 4 mL glacial acetic acid. Slides were dipped 10 times in toluene and mounted using mounting media (Sigma, Oakville, Québec, Canada). Slides were stored at room temperature prior microscopy analysis.

### Immunofluorescence (IF)

IF was performed on 7-μm thick sections subsequently fixed in 4% paraformaldehyde dissolved in phosphate buffer for 2 h at room temperature or overnight at 4°C. Sections were then washed, permeabilized in 1% Tween 20 for 10 min, blocked in 5% normal goat serum for 1 h, and then incubated with the primary rabbit anti-TDP43 antibody (1:500, Proteintech Group Inc., Chicago, IL, USA) for 16 h at 4°C. Sections were then washed and incubated for 1 h at room temperature in Alexa 488- or Alexa 598-conjugated anti-rabbit secondary antibodies diluted in blocking solution (1:500; Molecular Probes). Imaging was performed using an LSI 700 confocal microscope with Metamorph imaging software (Zeiss).

### Subcellular fractionation of TES and Western blotting

Whole protein lysate from reconstructed skin equivalents were extracted by cryogenic homogenization of the tissues using the CryoMill (Retsch) enabling grinding of tough, soft and elastic materials such as skin tissues into a fine recoverable powder. The cryomilled tissues were resolubilized in TNGT lysis buffer (50 mM Tris-HCl pH 7,4; 100 mM NaCl; 10% Glycerol; 1% Triton-X). The mix was then centrifuged at >20 000 rpm for 24 min at 4°C to separate supernatant (cytoplasmic fraction) and pellet (nuclear fraction). The nuclear pellet was then washed and resuspended in SUB lysis buffer (0,5% SDS, 8 M Urea, 2% b-mercapethanol in apyrogenic water). Total protein was quantified by the Lowry method and diluted in loading buffer (15% glycerol, 5% SDS, 80 mM Tris–HCl, pH 6.8, 5% β-mercapto-ethanol and 0.01% bromophenol blue). Each sample (25 μg) was run on a 14% SDS/glycine polyacrylamide gel and then transferred electrophoretically to a PVDF membrane (Biorad, Hercules, CA, USA). The blots were blocked in 5% non-fat milk/0.1% Tween 20 in phosphate-buffered saline (PBS) and probed with polyclonal anti-rabbit TDP-43 antibody (Proteintech Group Inc., Chicago, IL, USA) diluted 1:500 in the blocking buffer. Immunodetection was performed with a donkey anti-rabbit-HRP-labelled secondary antibody (1:1000, Thermo Scientific, Rockford, IL, USA), and the detection was performed with the ClarityTM Western ECL Substrate (Biorad, Hercules, CA, USA). The blots were then re-probed with an anti-b-actin antibody (1:6000, Abcam) to document equal loading.

## Results

### Structural abnormalities detected in ALS-TES by IHC

**Figure 1:**
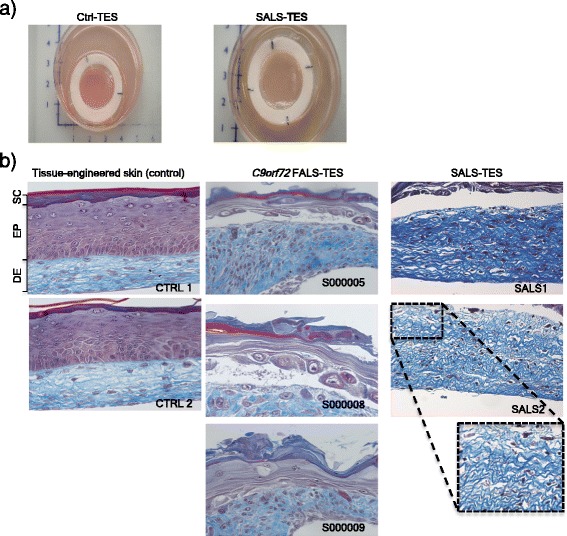
**Structural abnormalities detected in ALS-derived tissue engineered skins. a)** Macroscopic pictures of control-derived and ALS-derived tissue-engineered skins when cultured at the air-liquid interface. **b)** Masson’s trichrome colorations, specifically staining the dermis (DE) in blue and epidermis (EP) in purple, revealed a number of structural abnormalities including undifferentiated epidermis, abnormal dermo-epidermal junctions, delamination, abnormal collagen organization, keratinocyte infiltration and cohesive failure of the stratum corneum (SC) in both *C9orf72* FALS- and SALS-derived skins. In contrast control-derived reconstructed skins showed a well-developed and differentiated epidermis and highly organized dermis.

Masson’s trichrome is a special stain which is typically used to characterize and discriminate between various connective and soft tissue components. It is often utilized as the stain of choice of distinguishing histological changes in tumors, connective tissue diseases, muscle and fibroblast tumors, renal diseases and dermatology cases. Masson’s trichrome staining repeatedly revealed evident structural abnormalities uniquely detected in ALS-TES including an undifferentiated epidermis, cohesive failure of the stratum corneum, abnormal dermo-epidermal junction, delamination, keratinocyte infiltration, as well as collagen misorganization in both *C9orf72* FALS- and SALS-derived skins (Figure 1 and Additional file 4: Table S2). In contrast, control-derived tissue engineered skins showed a well-developed and differentiated epidermis and highly organized dermis.

### TDP-43 cytoplasmic inclusion detected in ALS-TES derived skin

**Figure 2:**
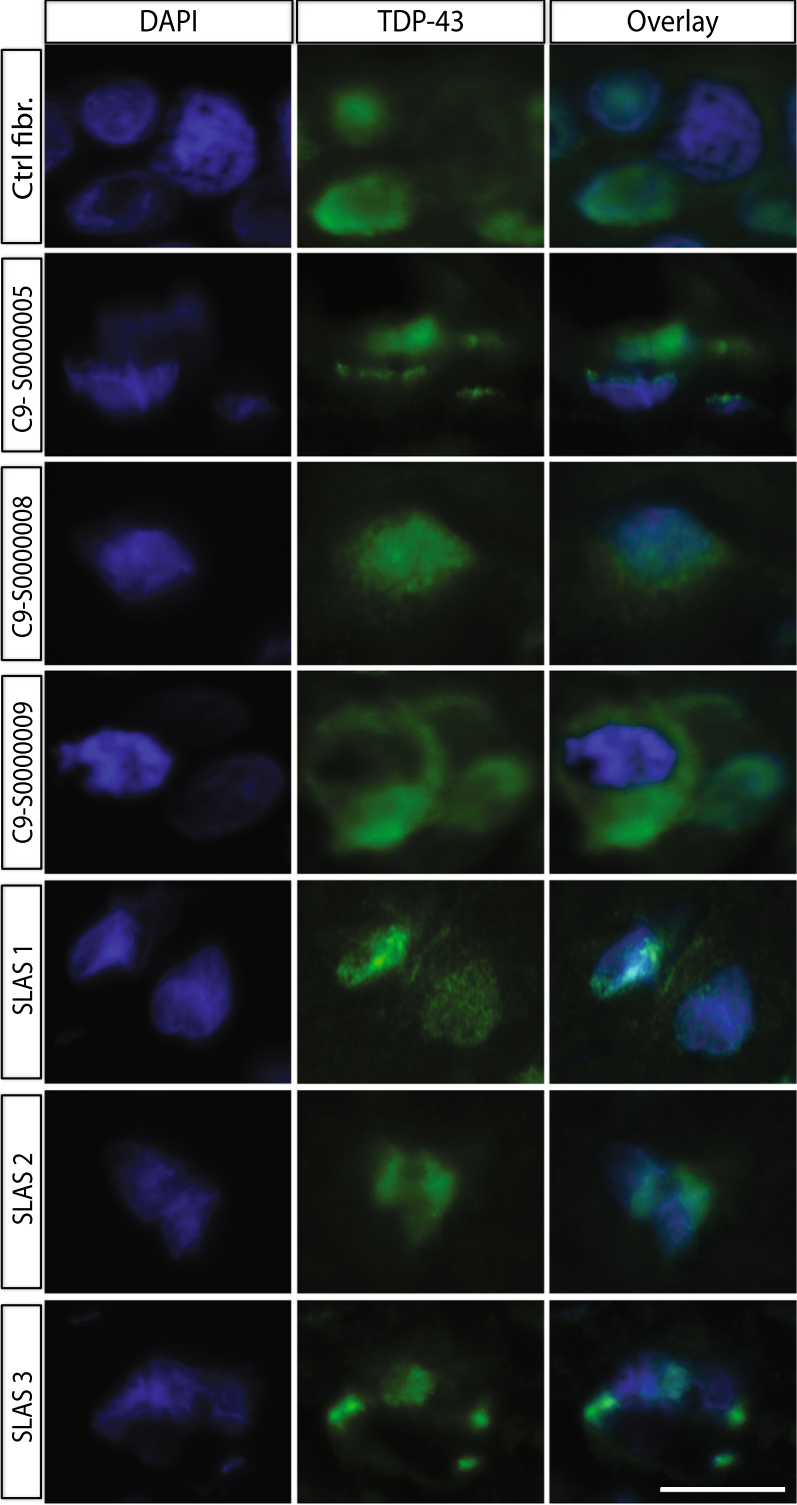
**Cytoplasmic TDP-43 accumulation detected in SALS- and C9orf72 FALS-derived tissue-engineered skins.** Indirect immunofluorescence analysis using anti-TDP43 antibody (green) counterstained with DAPI (blue) revealed cytoplasmic TDP-43 accumulation in SALS-derived as well as in C9orf72 FALS-derived tissue-engineered skins. Note that representative pictures of 7-um thick tissue-sections were stained and visualized using a standard epifluorescent microscope. Each picture was taken using the same microscope, camera and exposure settings. Scale bar (white): 10 μm.

**Figure 3:**
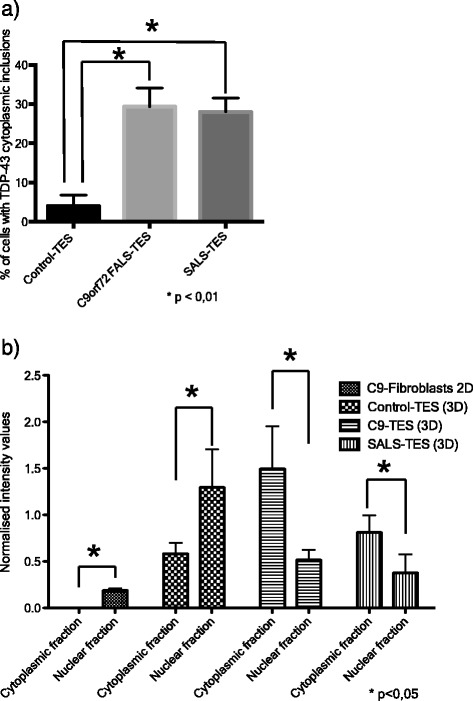
**Cellular counts and Western blots quantification of TDP-43 cytoplasmic accumulation. a)** Percentage of cell with positive cytoplasmic TDP-43 inclusions. 200 nuclei were counted for each of the generated tissue-engineered skins. *correspond to a *P* value < 0.01. **b)** Subcellular fractionation (cytoplasmic fraction vs nuclear fraction) of total protein extracted from ALS-fibroblast (2D culture), control-derived TES, C9ORF72-derived TES and SALS-derived TES (n = 5 for each group) was performed and loaded on a regular SDS-PAGE. TDP-43 expression in each fractionated sample was quantified using ImageJ after normalization against actin. Equal amount of proteins was used as shown on western blots after. *correspond to a *P* value < 0.05.

**Figure 4:**
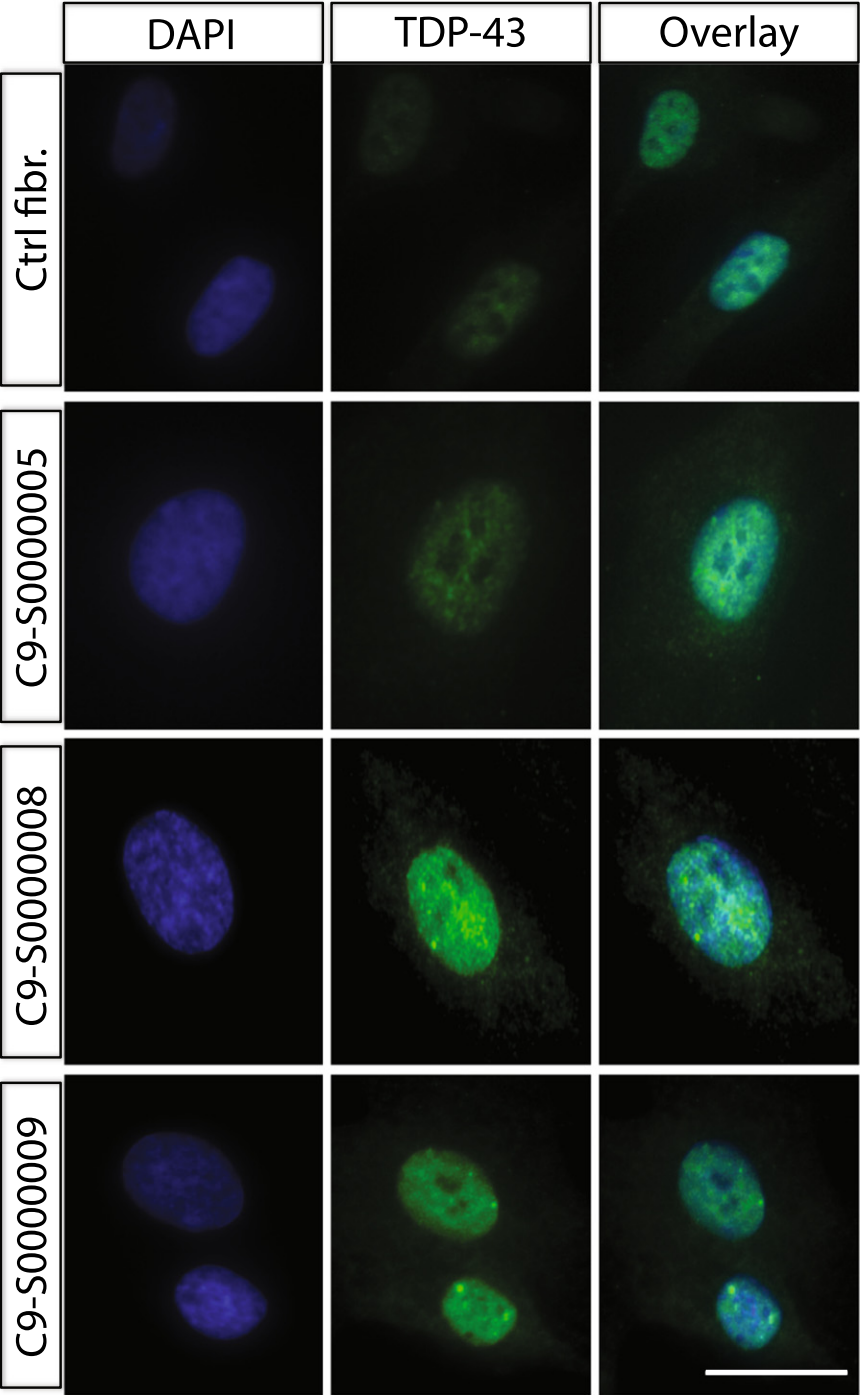
**Nuclear TDP-43 expression in** ***C9orf72*** **fibroblasts cultured cells.** Indirect immunofluorescence using anti-TDP43 commercial antibody (green) conterstained with DAPI (nucleus) revealed no cytoplasmic TDP-43 accumulation in *C9orf72* cultured fibroblasts collected from symptomatic and non-symptomatic *C9orf72* FALS patients. These results indicate that the described 3D tissue-engineered skin model, allowing for cell-to-cell and cell-to-matrix interactions, is necessary to observe the pathological cytoplasmic accumulation of TDP-43. Scale bar (white): 10 μm.

In order to determine if cytoplasmic TDP-43 aggregates can be detected in ALS-TES, 7-μm thick tissue sections were prepared and stained with commercial TDP-43 polyclonal antibody. Interestingly, TDP-43 cytoplasmic aggregates, characteristic of ALS pathology, were detected in SALS-derived skins by indirect immunofluorescence and standard microscopy (Figure 2). These results were also further confirmed by confocal microscopy, using 25-um thick sections (Additional file 5: Figure S3). To our knowledge, it is the first time that cytoplasmic TDP-43 aggregates are detected outside of the nervous system and in non-neuronal cells in any model so far. In contrast, no TDP-43 abnormal cytoplasmic accumulation was observed in control-derived reconstructed skin. To further confirm our results and determine if cytoplasmic TDP-43 can also be detected in non-symptomatic patients, we have generated *C9orf72* FALS-derived TES. Five out of six generated *C9orf72*-TES were derived from non-symptomatic patients carrying the GGGGCC DNA repeat expansion (Additional file 2: Figure S1; Table 1). Remarkably, cytoplasmic TDP-43 inclusions were detected by standard immunofluorescence analysis in both symptomatic and yet non-symptomatic *C9orf72*-linked ALS patients carrying the expansion (Figure 2). Actually, around 30% of the fibroblasts within the *C9orf72*- and SALS-derived skins presented cytoplasmic TDP-43 positive inclusions while only 4% of the fibroblasts in the control-derived skins demonstrated TDP-43 cytoplasmic inclusions (Figure 3A). Validation of these results were done by Western blotting after proper fractionation of the cytoplasmic and nulear fractions (Figure 3B; Additional file 6: Figure S4). Interestingly, cytoplasmic TDP-43 inclusions were only detected in our three dimensional (3D) ALS-TES model and were not detected in patient’s fibroblasts alone standard two dimensional (2D) cell culture indicating that our 3D skin model is necessary to observe the described phenotype (Figure 4). Western immunoblots, detected with nuclear (anti-nuclei antibody, clone 235-1, Millipore: cat# MAB1281) and cytoplasmic (anti-GAPDH antibody, AbD Serotec: cat# AHP1628) markers, revealed that our cytoplasmic fraction was completely free of nuclear protein indicating that the detected cytolasmic TDP-43 signal was not due to a contamination of the fraction with nuclear proteins (Additional file 6: Figure S4).

### TDP-43 cytoplasmic inclusion detected in native skin biopsies and in post-mortem CNS tissue collected from ALS patients

**Figure 5:**
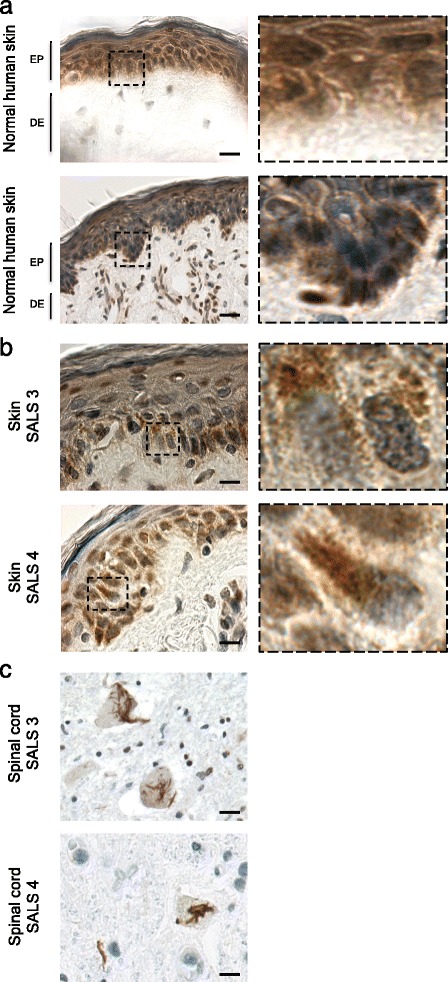
**Cytoplasmic TDP-43 detection in native skin biopsies and corresponding CNS tissues in SALS patients.** Immunohistochemistry analysis, using anti-TDP-43 antibody, revealed the presence of TDP-43 cytoplasmic accumulation in both native skin biopsies **(b)** and post-mortem **(c)** spinal cord tissues collected from SALS patients. Such TDP-43 cytoplasmic accumulation was not detected in control individuals **(a)**.

To further confirm our results, we undertook immunohistochemistry colorations, generally the method of choice in hospital pathological departments, on native skin biopsies collected from the corresponding ALS patients as well as on post-mortem spinal cord tissues when available. The IHC analysis confirmed the presence of cytoplasmic TDP-43 skein-like inclusions in the fibroblasts of the native skin biopsies taken from living SALS patients (Figure 5; Additional file 7: Figure S5). Moreover, round TDP-43 cytoplasmic inclusions in the dentate gyrus’s granular layer as well as skein-like TDP-43 cytoplasmic positive inclusions in neurons present in the ventral horn of the spinal cord were also detected by the neuropathologists after formal autopsies (Figure 5).

## Discussion

Here we report the generation and characterization of a novel *in vitro* tissue-engineered skin model to study ALS. Of particular interest, cytoplasmic TDP-43 inclusions, a well-established pathological feature of ALS, were uniquely seen in the ALS-derived TES as well as in native biopsies collected from the corresponding ALS patients. It has been previously shown that TDP-43 nuclear overexpression with no cytoplasmic inclusions can be detected in native skin biopsies collected from ALS patients [26]. It has also been shown that some aspects of TDP-43 proteinopathies, including increased TDP-43 nuclear expression and decreased motor neurons survival, can be detected in motoneuronally differentiated induced pluripotent stem cells [27,28]. However, we report here for the first time cytoplasmic TDP-43 accumulation in cells outside of the nervous system detected in both skin biopsies taken from ALS patients and in our patients-derived skin model. Interestingly, the TDP-43 mislocalization detected in our ALS-TES was observed relatively early in the disease process. They were in fact detected in the SALS-derived skins, long before the end stage of the disease, and in pre-symptomatic *C9orf72*-linked FALS patients carrying the GGGGCC hexanucleotide DNA expansion. Furthermore, our ALS-TES also presents a number of abnormal ECM-related features including epidermal undifferentiation, abnormal dermo-epidermal junction, delamination, keratinocyte infiltration and collagen disorganization. Interestingly, the cytoplasmic TDP-43 inclusions were also detected in CNS tissues by the neuropathologist after formal autopsies. Note also that the pre-symptomatic *C9orf72*-linked patients are still alive and have not yet developed any motor or dementia associated symptoms.

Although the detection of TDP-43 mislocalization in native skin biopsies presents a potential diagnostic marker for ALS and its progression, the use of skin biopsy limits the number of techniques used to validate the observed results. In contrast, the use of an *in vitro* tissue-engineered skin model presents a renewable source of human tissue allowing for the extensive testing required for the development of a novel *in vitro* validated diagnostic method. Our ALS-TES model would therefore provide a unique and innovative model to fully study the relation between skin changes and ALS, as well as identifying and validating specific ALS biomarkers for early diagnosis, to follow disease progression or to assess response to existing and future treatments. Such validated biomarkers, which can be detected outside the nervous system using easily accessible tissue, have never been described thoroughly in ALS. The development and application of more efficient, non- or minimally-invasive detection techniques in ALS has become crucial for biomarker-driven therapeutic discovery and also to monitor patient’s response to disease-modifying therapies.

Tissue engineering is a resolutely new scientific field combining cell culture advances with a better knowledge of the various extracellular matrix (ECM) components. Tissue-engineered models have numerous potential advantages over existing models, including cultivation in three dimensional (3D) geometries, which allows cell-cell as well as cell-ECM interactions and impact cell fate decisions, cell proliferation and survival, and other specialized functions [29]. It is well known that cells grown using a 3D cell culture technique more closely mimic natural tissues and organs than cells grown in 2D [30]. For instance, our results revealed that re-expression of disease phenotypes can be detected in the ALS-TES and not in standard 2D cell culture. Interestingly, genes associated with cell–matrix adhesion and cell–substrate adhesion were found to be misexpressed in SALS patients [31]. By extrapolation, we can imagine that the disruption of cell to matrix interaction detected in our ALS-TES could also reflect an ECM misorganization within the CNS, and adversely affect motor neurons, glia or other neurons. Cell–matrix interactions are critical for neuronal migration, which occurs primarily during development as neural progenitor cells migrate to their proper positions before differentiating [32]. Abnormal cell–matrix interactions could therefore alter the normal formation and wiring of the nervous system that could also lead to disease [33]. Disruption of cell-ECM interactions in the CNS could result in dysfunction of both neuronal and non-neuronal cell categories supporting the non-cell autonomous paradigm in ALS [15,18,20]. Accumulating evidence also indicates that matrix metalloproteinases (MMPs), able to degrade ECM proteins, are involved in the pathogenesis of a number of CNS disorders, and plays a major role in motor neuron degeneration both in patients and in different ALS mouse models [34-37]. The MMP genes are transcriptionally responsive to a wide variety of growth factors, cytokines, and reactive oxygen species. In the CNS, MMPs are synthesized by neurons, astrocytes, and microglia [38,39]. Although the cause of ALS remains obscure, potential roles of MMPs have been extensively investigated. In the developing CNS, MMPs are involved in neurogenesis, axonal guidance, and growth, myelinogenesis and angiogenesis. In the adult CNS they play a role in remodeling of the ECM, cell migration, and survival, in synaptic plasticity with an impact on learning and memory function, myelin turnover, and angiogenesis. Our study raises the question whether our ALS-TES may offer an easily accessible source of biomarkers that could allow monitoring specific aspects of disease pathology in ALS. Further studies investigating in parallel skin and neuronal tissue samples are necessary to confirm this hypothesis and to prove that the ECM-related abnormalities detected in our ALS-TES may also reflect systemic changes also present in the CNS.

## Conclusions

Since neurodegenerative diseases are becoming a serious concern for many countries, early therapy and disease prevention is essential. Diagnosis and management of neurodegenerative disorders such as Alzheimer’s disease, Parkinson’s disease, or ALS are currently central challenges in clinical neurology. While differing in clinical presentations, genetic predisposing factors or histopathological substrates, all these neurological disorders are characterized by progressive and relentless loss of neuronal cell populations within the CNS, leading to severe neurological deficits. While each neurodegenerative disorder has its own distinctive characteristics, it is well recognized that there is an overlap between various disorders, both in clinical presentations and in histopathological features including skin changes over the disease course [22]. Conceivably, the application of tissue-engineered skin models to facilitate the identification of biomarkers for early diagnosis and disease progression will become highly attractive and will be of high importance for the future research, allowing monitoring of patients longitudinally. Such cross-disease biomarkers are currently not available in clinical neurological practice. However, our study is the first to show that TDP-43 and ECM-related pathology can be reproducibly analyzed using biopsies from a peripheral tissue in living patients. To validate the present findings and to strengthen our results, a larger sample of ALS and control subjects will first be required. Then, correlations between the increased TDP-43 expression and/or mislocalization found in our ALS-TES, and disease severity, survival, age of onset and progression could then be determined in order to develop of an early diagnostic/prognostic test for ALS. Hopefully, routine skin biopsies will become a useful tool for ante-mortem neuropathological diagnosis of ALS and other neurodegenerative diseases, and also provides insight into the progression of motor and non-motor symptoms. The use of the skin biopsies and/or tissue-engineered skins as a window into the CNS represents an original approach with implications that may well extend beyond ALS.

## Additional files

Additional file 1: Table S1. Revised El Escorial criteria for diagnosing ALS. Additional file 2: Figure S1. Familial *C9orf72*-linked ALS pedigree. Genotypes of analyzed family members are indicated (Squares denote males, circles-females, black symbols- affected individuals, symbols with central dot-unaffected mutation carriers, slash marks-deceased individuals). The numbers to the upper right of individual family members indicate age at death/current age). Additional file 3: Figure S2. Tissue-engineered skin equivalent using self-assembly method. Cultured fibroblasts at passage 3, grown in DMEM (Dulbecco-Vogt modification of Eagle's medium) (Invitrogen, Burlington, ON, Canada) supplemented with 10% Fetal Calf Serum (FCS) (Invitrogen), 100 IU/ml penicillin G (Sigma, Oakville, QC, Canada) and 25 μg/ml gentamicin (Schering, Pointe-Claire, QC, Canada) in 8% CO2 at 37°C, at passage five were seeded at a concentration of 3 × 10^4^ cells/cm^2^ on tissue culture dishes. At confluence, cultured media was supplemented with 50 μg/ml of ascorbic acid (Sigma) for 20 days in order to induce secretion of extracellular matrix proteins and to form a fibroblast sheet. Three fibroblast sheets are superimposed for 3 additional days to allow sheet adhesion. Epithelial cells (isolated keratinocytes), were cultured in a combination of Dulbecco-Vogt modification of Eagle's medium with Ham's F12 (3:1) supplemented with 5% Fetal Clone II serum (Hyclone, Scarborough, Ontario, Canada), 5 μg/mL insulin (Sigma Oakville, Canada), 0.4 μg/mL hydrocortisone (Calbiochem, EMD Biosciences, Gibbstown, NJ), 10 − 10 M cholera toxin (MP Biomedicals, Montréal, Québec, Canada), 10 ng/mL human epidermal growth factor (Austral Biological, San Ramon, CA), 100 IU/mL penicillin G (Sigma), and 25 μg/mL gentamicin (Schering), are seeded on top of the mature fibroblast sheets at a concentration of 8 × 10^3^ cells/cm^2^ . The reconstructed skin equivalent is then cultivated at the air-liquid interface in order to enhance formation of the stratum corneum (outermost layer of the epidermis). The duration of each maturation phases is 7 days. Additional file 4: Table S2. Abnormal structural skin features detected in ALS-tissue engineered skin. Additional file 5: Figure S3. Cytoplasmic TDP-43 accumulation detected in SALS-derived tissue-engineered skins. Indirect immunofluorescence and confocal analysis using anti-TDP43 antibody (green) counterstained with DAPI (blue) revealed cytoplasmic TDP-43 accumulation and an obvious increase in TDP-43 expression specifically in SALS-derived skins. Note that representative pictures of 50-um thick tissue-sections were stained and visualized using a confocal microscopy, taken from one Control- and SALS-derived skins are illustrated. Each picture was taken using the same microscope, camera and exposure settings. Additional file 6: Figure S4. Original Western blots and specificity of the protein fractionation method. A) Western blot analysis revealed that, using anti-nuclei (nuclear fraction) anti-GAPDH antibody (mainly cytoplasmic fraction) antibodies, revealed that our cytoplasmic fraction was completely free of nuclear protein. This clearly indicates that the detected cytolasmic TDP-43 signal was not due to a contamination of the fraction with nuclear proteins. B) Original Western blots, immunostained with anti-TDP43 and anti-actin antibodies, used to quantify the normalized TDP-43 signal. Additional file 7: Figure S5. Cytoplasmic TDP-43 inclusions are detected in the fibroblast cells of SALS native skins. Indirect immunofluorescence analysis, using anti-TDP-43 (red) and anti-vimentin (green), counterstained with DAPI (blue), confirmed that the TDP-43 mislocalization is detected in fibroblast cells at the dermo-epidermal junction and in the dermis of SALS native skins (white arrows).
