# Supplementary material for: Early detection of structural abnormalities and cytoplasmic accumulation of TDP-43 in tissue-engineered skins derived from ALS patients
Source: Acta Neuropathol Commun. 2015 Jan 31;3:5. doi: 10.1186/s40478-014-0181-z (PMC4359444; doi:10.1186/s40478-014-0181-z)
Supplement: Additional file 1: Table S1. — Revised El Escorial criteria for diagnosing ALS. [file 40478_2014_181_MOESM1_ESM.docx]

**Additional table 1: Revised El Escorial criteria for diagnosing ALS**

| **ALS diagnostic category** | **Requirements** |
| --- | --- |
| Definite ALS | LMN and UMN signs in 3 regions of the body |
| Definite familial ALS | LMN and UMN signs in 1 region of the body plus laboratory-supported identification of gene mutation associated with ALS |
| Probable ALS | LMN and UMN signs in 2 regions of the body (some UMN signs rostral to LMN signs) |
| Probable ALS (laboratory supported) | LMN and UMN signs in 1 region of the body plus electromyographic evidence of acute denervation in 2 or more muscles in 2 or more limbs |
| Possible ALS | LMN and UMN signs in 1 region of the body |

ALS – amyotrophic lateral sclerosis, LMN – lower motor neuron, UMN – upper motor neuron.
