# Supplementary figures and images for: Early detection of structural abnormalities and cytoplasmic accumulation of TDP-43 in tissue-engineered skins derived from ALS patients
Source: Acta Neuropathol Commun. 2015 Jan 31;3:5. doi: 10.1186/s40478-014-0181-z (PMC4359444; doi:10.1186/s40478-014-0181-z)

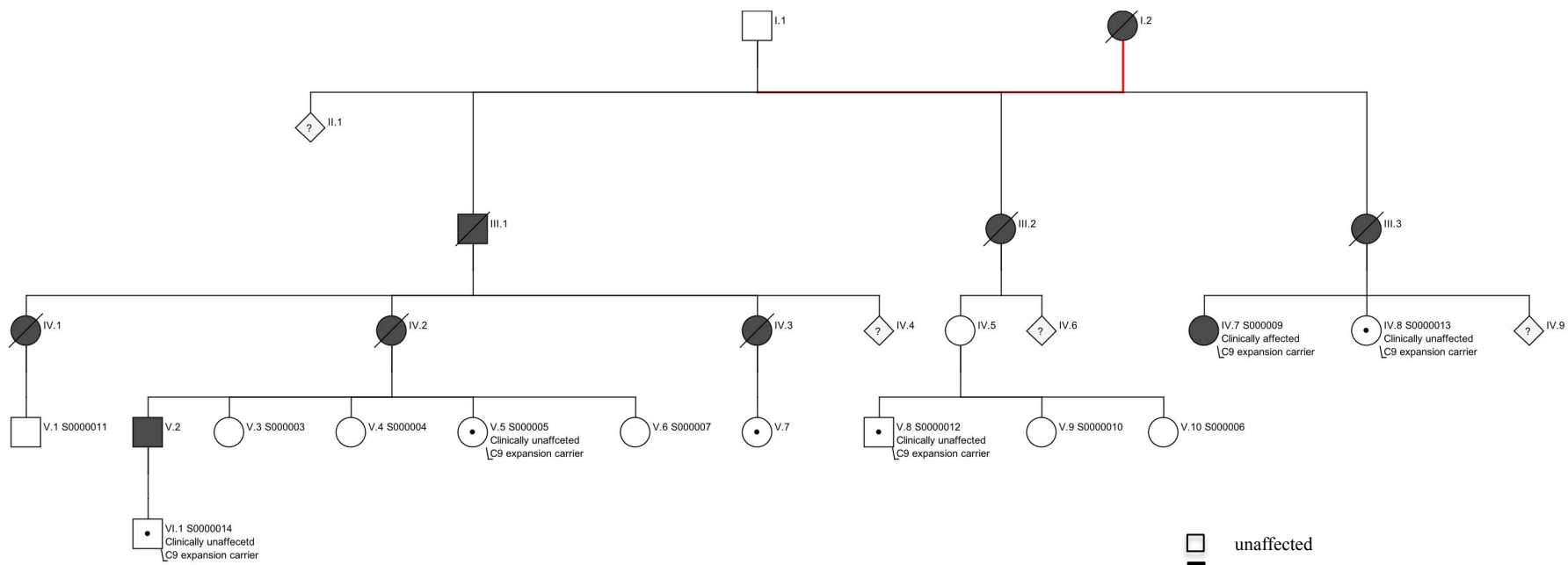

Supplement: Additional file 2: Figure S1. — Familial C9orf72-linked ALS pedigree. Genotypes of analyzed family members are indicated (Squares denote males, circles-females, black symbols- affected individuals, symbols with central dot-unaffected mutation carriers, slash marks-deceased individuals). The numbers to the upper right of individual family members indicate age at death/current age). [file 40478_2014_181_MOESM2_ESM.pdf]

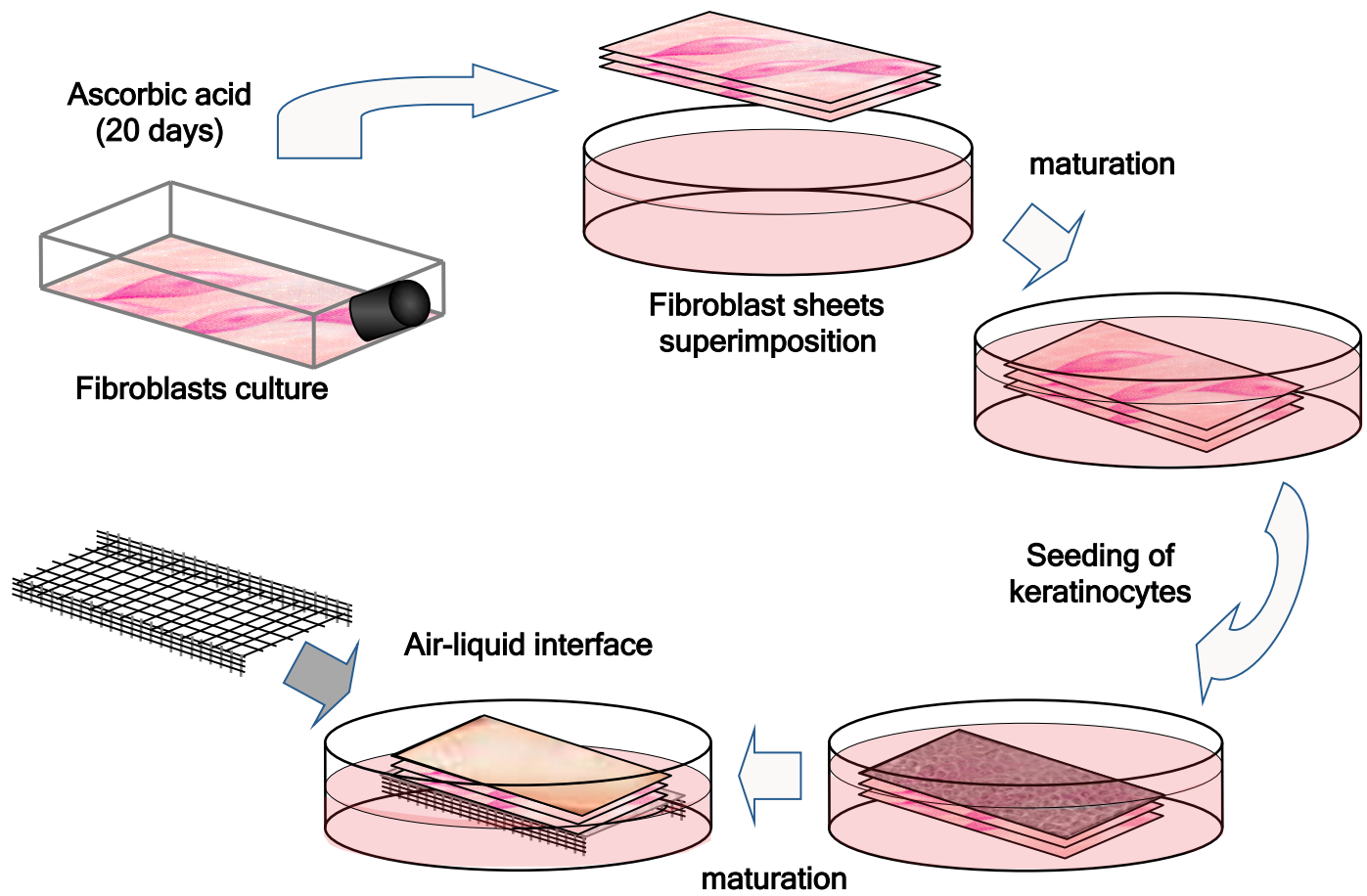

Supplement: Additional file 3: Figure S2. — Tissue-engineered skin equivalent using self-assembly method. Cultured fibroblasts at passage 3, grown in DMEM (Dulbecco-Vogt modification of Eagle's medium) (Invitrogen, Burlington, ON, Canada) supplemented with 10% Fetal Calf Serum (FCS) (Invitrogen), 100 IU/ml penicillin G (Sigma, Oakville, QC, Canada) and 25 μg/ml gentamicin (Schering, Pointe-Claire, QC, Canada) in 8% CO2 at 37°C, at passage five were seeded at a concentration of 3 × 10^4^ cells/cm2 on tissue culture dishes. At confluence, cultured media was supplemented with 50 μg/ml of ascorbic acid (Sigma) for 20 days in order to induce secretion of extracellular matrix proteins and to form a fibroblast sheet. Three fibroblast sheets are superimposed for 3 additional days to allow sheet adhesion. Epithelial cells (isolated keratinocytes), were cultured in a combination of Dulbecco-Vogt modification of Eagle's medium with Ham's F12 (3:1) supplemented with 5% Fetal Clone II serum (Hyclone, Scarborough, Ontario, Canada), 5 μg/mL insulin (Sigma Oakville, Canada), 0.4 μg/mL hydrocortisone (Calbiochem, EMD Biosciences, Gibbstown, NJ), 10 − 10 M cholera toxin (MP Biomedicals, Montréal, Québec, Canada), 10 ng/mL human epidermal growth factor (Austral Biological, San Ramon, CA), 100 IU/mL penicillin G (Sigma), and 25 μg/mL gentamicin (Schering), are seeded on top of the mature fibroblast sheets at a concentration of 8 × 10^3^ cells/cm2 . The reconstructed skin equivalent is then cultivated at the air-liquid interface in order to enhance formation of the stratum corneum (outermost layer of the epidermis). The duration of each maturation phases is 7 days. [file 40478_2014_181_MOESM3_ESM.pdf]

TDP-43; DAPI

Ctrl

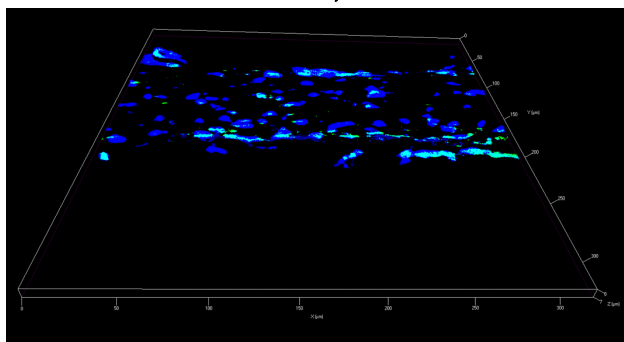

SALS 1

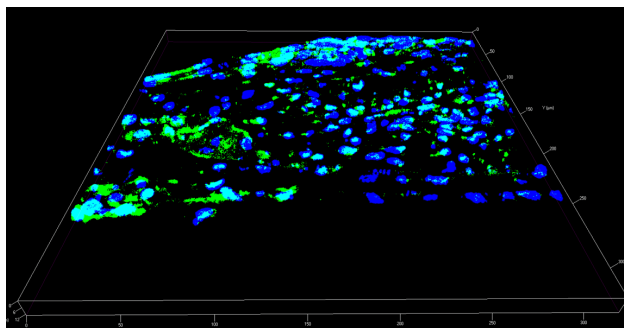

Supplement: Additional file 5: Figure S3. — Cytoplasmic TDP-43 accumulation detected in SALS-derived tissue-engineered skins. Indirect immunofluorescence and confocal analysis using anti-TDP43 antibody (green) counterstained with DAPI (blue) revealed cytoplasmic TDP-43 accumulation and an obvious increase in TDP-43 expression specifically in SALS-derived skins. Note that representative pictures of 50-um thick tissue-sections were stained and visualized using a confocal microscopy, taken from one Control- and SALS-derived skins are illustrated. Each picture was taken using the same microscope, camera and exposure settings. [file 40478_2014_181_MOESM5_ESM.pdf]

TDP-43

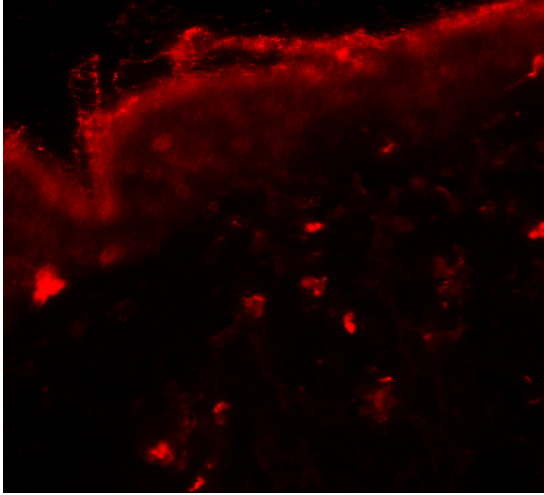

Vimentin

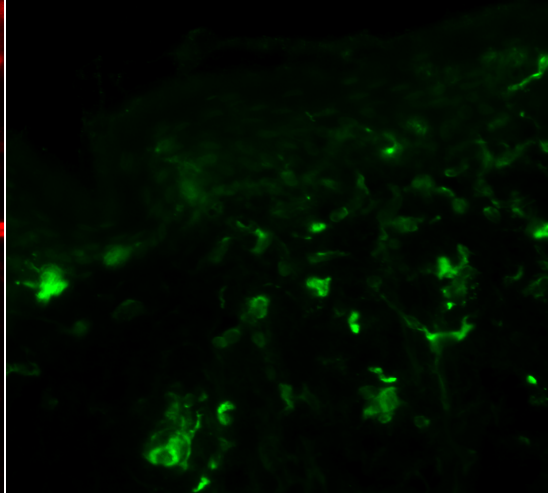

DAPI

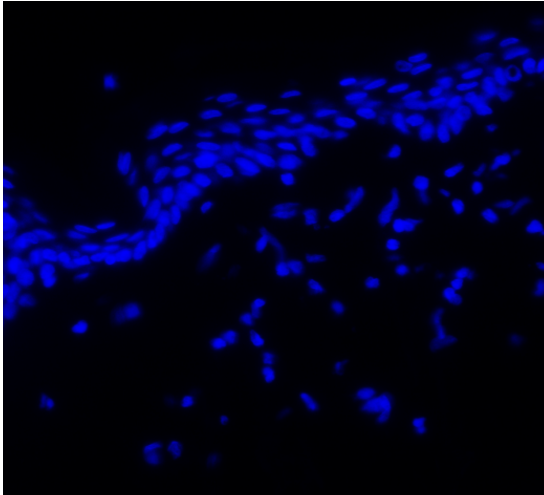

Overlay

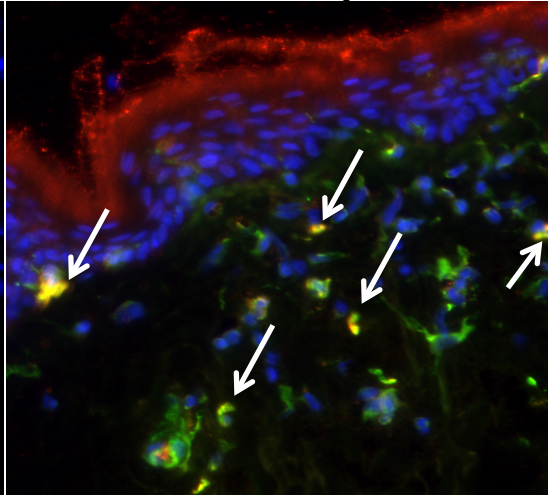

Supplement: Additional file 7: Figure S5. — Cytoplasmic TDP-43 inclusions are detected in the fibroblast cells of SALS native skins. Indirect immunofluorescence analysis, using anti-TDP-43 (red) and anti-vimentin (green), counterstained with DAPI (blue), confirmed that the TDP-43 mislocalization is detected in fibroblast cells at the dermo-epidermal junction and in the dermis of SALS native skins (white arrows). [file 40478_2014_181_MOESM7_ESM.pdf]
