# Supplementary material for: Early detection of structural abnormalities and cytoplasmic accumulation of TDP-43 in tissue-engineered skins derived from ALS patients
Source: Acta Neuropathol Commun. 2015 Jan 31;3:5. doi: 10.1186/s40478-014-0181-z (PMC4359444; doi:10.1186/s40478-014-0181-z)
Supplement: Additional file 4: Table S2. — Abnormal structural skin features detected in ALS-tissue engineered skin. [file 40478_2014_181_MOESM4_ESM.pdf]

Additional Table 2: Abnormal structural skin features detected in ALS-tissue engineered skin

ID number	Undifferenciated epidermis	Cohesive failure of the *stratum corneum*	Abnormal dermo-epidermal junction	Delamination	Keratinocyte infiltration	Collagen misornization
SALS 1	x	x	x	x	x	x
SALS 2	x	x	x	x		x
SALS 3	x		x	x		x
SALS 4	x	x	x	x		x
SALS 5	x		x	x	x	x
SALS 6	x		x	x	x	x
C9-S000005	x	x	x	x	x	x
C9-S000008	x	x	x	x	x	x
C9-S000009	x	x	x	x	x	x
C9-S000012	x	x	x	x	x	x
C9-S000013	x	x	x	x	x	x
C9-S000014	x	x	x	x	x	x
