# Supplementary material for: Early detection of structural abnormalities and cytoplasmic accumulation of TDP-43 in tissue-engineered skins derived from ALS patients
Source: Acta Neuropathol Commun. 2015 Jan 31;3:5. doi: 10.1186/s40478-014-0181-z (PMC4359444; doi:10.1186/s40478-014-0181-z)
Supplement: Additional file 6: Figure S4. — Original Western blots and specificity of the protein fractionation method. A) Western blot analysis revealed that, using anti-nuclei (nuclear fraction) anti-GAPDH antibody (mainly cytoplasmic fraction) antibodies, revealed that our cytoplasmic fraction was completely free of nuclear protein. This clearly indicates that the detected cytolasmic TDP-43 signal was not due to a contamination of the fraction with nuclear proteins. B) Original Western blots, immunostained with anti-TDP43 and anti-actin antibodies, used to quantify the normalized TDP-43 signal. [file 40478_2014_181_MOESM6_ESM.pdf]

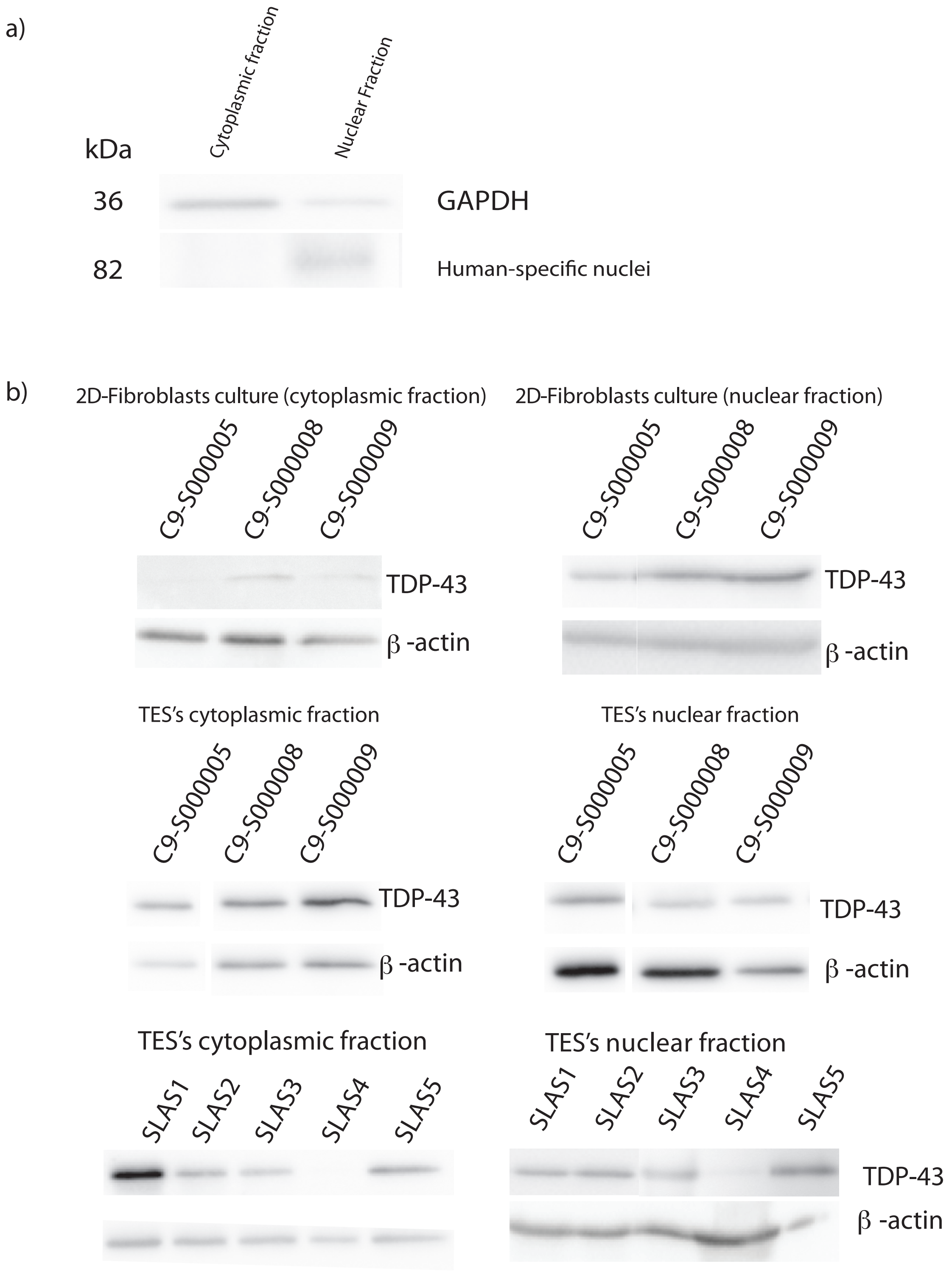
a)
Cytoplasmic fraction
Nuclear Fraction
kDa
36
GAPDH
82
Human-specific nuclei
b)
2D-Fibroblasts culture (cytoplasmic fraction)
2D-Fibroblasts culture (nuclear fraction)
C9-S000005
C9-S000008
C9-S000009
TDP-43
β -actin
TES's cytoplasmic fraction
TES's nuclear fraction
SLAS1
SLAS2
SLAS3
SLAS4
SLAS5
